# A novel antibacterial biomaterial mesh coated by chitosan and tigecycline for pelvic floor repair and its biological performance

**DOI:** 10.1093/rb/rbaa034

**Published:** 2020-09-02

**Authors:** Changyan Liang, You Ling, Feng Wei, Lijie Huang, Xiaomao Li

**Affiliations:** r1 Department of Gynecology and Obstetrics, Third Affiliated Hospital of Sun Yat-Sen University, Guangzhou, Guangdong, China; r2 National Engineering Laboratory for Regenerative Medical Implant Devices, Guanhao Biotech Group, Guangzhou Juming Biotech Co., Ltd, Guangzhou, Guangdong, China; r3 Department of Neurology, Guangdong Second Provincial General Hospital, Guangzhou, Guangdong, China

**Keywords:** chitosan nanoparticles, tigecycline, extracellular matrix, antibacterial membrane, pelvic floor repair

## Abstract

The biomaterials composed of mammalian extracellular matrix (ECM) have a great potential in pelvic floor tissue repair and functional reconstruction. However, bacterial infection does cause great damage to the repair function of biomaterials which is the major problem in clinical utilization. Therefore, the development of biological materials with antimicrobial effect is of great clinical significance for pelvic floor repair. Chitosan/tigecycline (CS/TGC) antibacterial biofilm was prepared by coating CS/TGC nanoparticles on mammalian-derived ECM. Infrared spectroscopy, scanning electron microscopy, bacteriostasis circle assay and static dialysis methods were used to characterize the membrane. MTS assay kit and DAPI fluorescence staining were used to evaluate cytotoxicity and cell adhesion. The biocompatibility was assessed by subabdominal implantation model in goats. Subcutaneous antimicrobial test in rabbit back was used to evaluate the antimicrobial and repairing effects on the infected wounds *in vivo*. Infrared spectroscopy showed that the composite coating had been successfully modified. The antibacterial membrane retained the main structure of ECM multilayer fibers. *In vitro* release of biomaterials showed sustained release and stability. *In vivo* studies showed that the antibacterial biological membrane had low cytotoxicity, fast degradation, good compatibility, anti-infection and excellent repair ability.

## Introduction

Pelvic organ prolapse (POP) is a major health problem for women [1]. The prevalence of pelvic organ prolapse increases with age [2]. It is estimated that 50% of women over 50 years old have pelvic organ prolapse [3, 4]. Reconstruction of pelvic floor with mesh is the main surgical method for POP [5]. Compared with synthetic material mesh, biomaterial mesh has the advantages of good biomechanical compliance and excellent biocompatibility [6].

Mammalian extracellular matrix (ECM) biomaterials have showed great potential in pelvic floor tissue repair and functional reconstruction [7, 8]. However, bacterial infection that destroys the function of biomaterials is one of the major problems [9, 10]. Once infected by bacteria, biomaterial itself becomes the nutrient matrix for bacterial growth and reproduction, which will lead to the implant falling off but difficult to take out after surgery failure.

Chitosan (CS) has been widely used in biomedical field [11]. It is a kind of nontoxic, biodegradable and renewable material, which has no pollution to the environment and exhibits excellent adsorption performance [12, 13]. There are many positive charges on the surface of CS, which is one of the reasons why CS has antibacterial and fungistatic properties [14, 15].

Tigecycline (TGC) is the first clinical intravenous glycycline approved by the Food and Drug Administration (FDA) [16], and it has been modified to kill tetracycline-resistant bacteria [17]. TGC can block the entry of tRNA by binding with 30S ribosome of bacteria, which makes amino acids unable to bind peptide chain, and finally blocks the synthesis of bacterial protein and restricts the growth of bacteria [18].

A potential solution to improve the antibacterial ability of the mammalian ECM biomaterials is to combine antibacterial material and drug into the biomaterials. In this respect, CS and TGC are attractive for their known safety profile and antibacterial property. The purpose of this study is to develop a new type of antimicrobial drug-loaded sustained-release composite biomaterial mesh and evaluate its antimicrobial effect, *in vitro* cytotoxicity, biocompatibility and *in vivo* antibacterial activity.

## Materials and methods

### Preparation of precursor suspension and construction of drug-loaded antibacterial membrane

At first, we prepared 100 µg/µl TGC solution as follows: accurately weighed 10 mg TGC (Sigma), dissolved it in 100 µl water and stored it in the dark. TGC solutions of 25 and 50 µg/µl were prepared by the same method.

Then 10 µg/µl CS solution was prepared: 100 mg CS (Zhejiang Nantong Xingcheng Biotechnology Co. Ltd, China) was dissolved in 10 ml 1% acetic acid solution. After that 100 µg/µl TGC solution and 0.1% triphosphate (TPP) solution was added and mixed for 2 h. The mass ratio of TPP and CS was 1:5 [19]. CS molecules were coated with tegacycline, self-coagulated to form nanoparticles and then form macromolecules and nanoparticles suspension with original CS solution.

The mammalian-derived ECM (Guanhao Biotechnology Co. Ltd, Guangdong Province, China) treated with patent technology (US 6106555) was cut into a fixed-size membrane. The nanodrug-loaded precursor suspension was modified on the surface of the 3D interlaced micro nanocollagen fibers in membrane structure, which was incubated for 2–6 h, prefrozen for 24 h at −40°C and freeze-dried for 24 h at low temperature. Then CS/TGC antibacterial biological membranes were obtained.

After 10 ml 10 µg/µl CS solution and 100 µl 25 µg/µl TGC mixed, CS/TGC membrane-1 (25 μg/μl TGC) is fabricated in the same way as CS/TGC membrane.

After 10 ml 10 µg/µl CS solution and 100 µl 50 µg/µl TGC mixed, CS/TGC membrane-2 (50 µg/µl TGC) is fabricated in the same way as CS/TGC membrane.

Taking the pure ECM membrane as the control, CS/TGC antibacterial biological membranes were detected by infrared spectroscopy (Tensor 27 Ftir Spectrometer, Bruker Company, Germany) and SEM (LEO 1530VP LEO 1530 field emission scanning electron microscope, Germany).

### Determination of antimicrobial effect of nanodrug-loaded biological membrane

In this study, *Escherichia coli* (ATCC 25922, 1.5 × 10^8^ CFU/ml) was used as the representative of Gram-negative bacteria and *Staphylococcus aureus* (ATCC 25933, 1.5 × 10^8^ CFU/ml) as the representative of Gram-positive bacteria to preliminarily evaluate the antimicrobial activity of the biological membrane.

The ECM and antimicrobial membranes were separately placed in the center of the plate containing the mentioned bacteria. After incubated at 37°C for 18–24 h, the diameter of the surrounding inhibition zone (mm) was accurately measured by Vernier caliper. After 24, 48, 72 and 96 h of culture, the diameter of the inhibition zone was measured. When the diameter of bacteriostatic circle was more than 7 mm, the antibacterial activity was considered to be effective. All the experiments were repeated three times.

### Determination of drug sustained release of the biological membrane

TGC was accurately weighed and dissolved in 70% methanol solution to establish standard solutions of 4000, 1000, 100, 10 and 1 µg/ml. The TGC sustained-release property of the antimicrobial membrane was determined by high-performance liquid chromatography (HPLC; Agilent, Germany), accurately weighed 50 mg antibacterial biological membrane materials, dispersed them in 25 ml phosphate-buffered saline (PBS) (pH = 7.4) solution and put them into dialysis bag (cut-off molecular weight = 3400). The dialysis bag clamped at both ends was then suspended in a conical flask containing 200 ml PBS and placed on a constant temperature shaker to vibrate. The control temperature was (37 ± 0.5)°C, and the rotating speed was 120 R/min. Samples were taken out at 4 h, 1, 2, 3, 5, 7, 9, 12, 15 and 22 days, respectively. At the same time, the same amount of fresh PBS solution at the same temperature was added and the vibration continued.

After high-speed centrifugation of the sample, the supernatant was taken and the 0.22 μm organic filter was used to obtain the clear solution. The peak area of TGC at different time points was measured by HPLC, and the release concentration and cumulative drug release rate were calculated. TGC soaking membrane without CS modification was taken as the control group and repeated the above steps. In order to verify the stability of drug sustained release, a static drug content determination method was used for comparative observation. All measurements were performed in triplicate, and the data were reported as means ± SD.

### Determination of cytotoxicity and cell adhesion of the biological membrane

Fibroblast L929 cell line derived from ATCC (Manassas, VA) was cultured in Dulbecco's Modified Eagle Medium (DMEM) with 10% fetal bovine serum and 100 U/ml penicillin-streptomycin under 37°C, 5% CO_2_ for the *in vitro* study.

The biological membrane was totally immersed in DMEM at 37°C for 24 h and the supernatant was collected. The supernatant was added to 96-well plate inoculated with L929 cells and cultured for 48 or 72 h. Cells with fresh medium were used as negative control group, and those with 5% dimethyl sulfoxide were used as positive control group. The optical density values of negative control, positive control, ECM membrane and CS/TGC antibacterial biological membrane were measured with the MTS test kit (Sigma) on the microplate reader.

Cell adhesion of the biological membrane was determined by 4,6-Diamidino-2-phenylindole (DAPI) staining. Cells were seeded on the surface of antibacterial biofilm and cultured for 1, 4, 7 and 14 days. After discarding the culture medium, cells was fixed with polyoxymethylene at room temperature and incubated with DAPI fluorescent staining solution for 30 min. DAPI stained cells were blue under inverted fluorescent microscope.

### Detection of biocompatibility by *in vivo* implantation

Animal experiments had been approved by our institutional ethics committee. Eight female goats (18–20 kg) were purchased from the experimental animal center of Guanhao Biotechnology Co. Ltd. After disinfection and anesthesia, the incision was made in the left and right lower abdomen of the goats, and the skin, subcutaneous tissue, muscle and fascia were cut layer by layer until the peritoneum was exposed. Bard mesh (purchased from C. R. Bard, Inc.), domestic mesh (purchased from Condiner Medical Technology Co. Ltd), drug-loaded antimicrobial membrane and pure ECM membrane were, respectively, placed between rectus sheath and peritoneum. Goats were executed humanely 3 and 6 months after implantation. The appearance of implant site and the integration of the meshes and normal tissue were observed. The peripheral tissue samples were collected for histopathological analysis and MMP9 immunohistochemical assay.

### Detection of antibacterial activity by *in vivo* infection

This animal experiment was also approved by our institutional ethics committee. Fifteen New Zealand female white rabbits (aged 5 months, 3–3.5 kg) were purchased from the experimental animal center of Guanhao Biotechnology Co. Ltd. After disinfection and anesthesia, four 2 × 2 cm wounds were evenly made on the back of the rabbit, and the whole skin was excised until the muscle was exposed. The biological membrane was placed on the surface of the wound and 0.5 ml 2 × 10^5^ CFU/ml bacterial solution was dripped on the membrane to make the infection model. The biological membrane was fixed with suture, and the wound was covered with dressing. The changes of infection foci and the skin healing pattern were recorded and analyzed at different time points.

### Statistical analysis

Data were analyzed by SPSS version 20.0 software package (SPSS, IL). One-way analysis of variance with post-hoc analysis (Bonferroni) was used for multiple comparisons. *P *<* *0.05 was considered to be statistically significant.

## Results

### Construction of the drug-loaded antibacterial membrane

**Figure 1.**
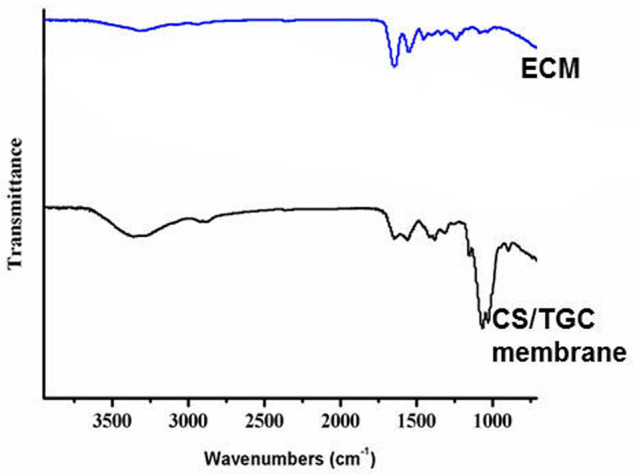
Infrared spectrogram of ECM membrane and CS/TGC membrane

Compared with pure ECM membrane, the absorption peak of CS composite antibacterial membrane widened at around 3359 cm^−1^ (Fig. 1). This belonged to O–H and N–H stretching vibration and was the characteristic adsorption peak by the overlapping of intramolecular and intermolecular hydrogen bonds of CS compound. Around 1647 cm^−1^ was the absorption peak of C=O stretching vibration, 1559 cm^−1^ was the absorption peak of N–H in-plane bending vibration and 1378 cm^−1^ was the absorption peak of N–H out of plane bending vibration [20]. About 1152 cm^−1^ was the asymmetrical stretching vibration peak of C–O–C oxygen bridge, whereas 1068 and 1028 cm^−1^ peak represented the stretching vibration of sugar ring. These strong absorption peaks were the characteristic of CS compound and were not detectable in the absorption spectra of pure ECM membranes (Fig. 1) which indicated that CS was successfully coated on the ECM biological membrane.

**Figure 2.**
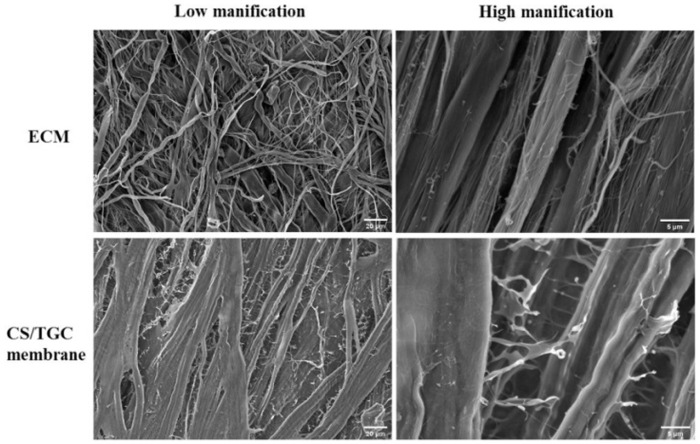
SEM of ECM and CS/TGC membrane in low magnification (500×) and high magnification (2500×), accelerated voltage: 10 kV, working distance: 11.7 mm (ECM), 5.9 mm (CS/TGC membrane)

ECM was mainly composed of multilayer orderly arranged filaments, which had different orientations, and thus formed a 3D space grid porous structure (Fig. 2). The CS/TGC membrane preserved the similar main structure (Fig. 2): multi-layer orderly arranged filaments with fluffy and scaly coating. Though the diameter of the 3D holes on membrane surface became smaller, the connectivity still maintained. In addition, compared with the ECM membrane, the sentus like micro/nanostructures formed by CS and TGC were observed on the CS/TGC membrane fibers (Fig. 2).

### Antimicrobial properties of drug-loaded antimicrobial membrane

**Figure 3.**
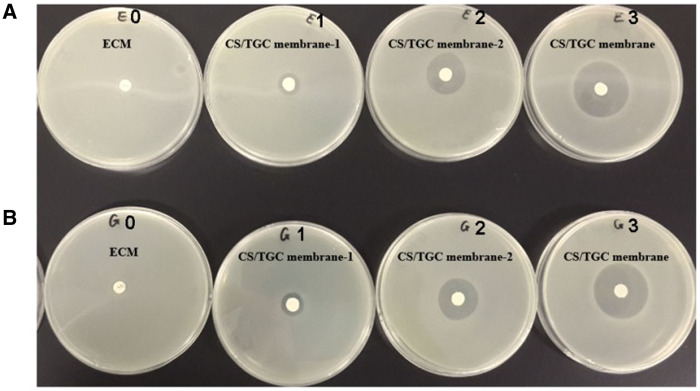
Antibacterial assessment of CS/TGC membrane. (A) The antibacterial ability of different groups against *Escherichia coli* in 1 day. (B) The antibacterial ability of different groups against *Staphylococcus aureus* in 1 day

After 24 h of cultivation of *E. coli* and *S. aureus*, the bacteria in ECM group grew well without the appearance of bacteriostasis circle and showed no obvious antimicrobial activity (Fig. 3). However, in the nanodrug antimicrobial membrane group, there were obvious antibacterial circles at the dosage of 25, 50, 100 µg/µl TGC in 1 day, and with the increase of the concentration of antibacterial agents, the diameter of the inhibition zone increased significantly, showing good antimicrobial performance (Fig. 3). The size of inhibition zone of biologic antimicrobial materials in different culture time is provided in Table 1.

**Table 1.** The size of inhibition zone of biologic antimicrobial materials in different culture time

TGC concentration		25 µg/µl (mm)	50 µg/µl (mm)	100 µg/µl (mm)
*Escherichia coli*	24 h	9.88	14.54	30.65
	48 h	9.87	14.44	30.63
	72 h	9.63	14.48	30.23
	96 h	9.14	14.43	30.25
*Staphylococcus aureus*	24 h	10.21	16.31	29.67
	48 h	10.18	16.28	29.65
	72 h	10.13	16.26	29.58
	96 h	10.05	16.22	29.57

### Observation on drug release mode of drug-loaded antimicrobial membrane

**Figure 4.**
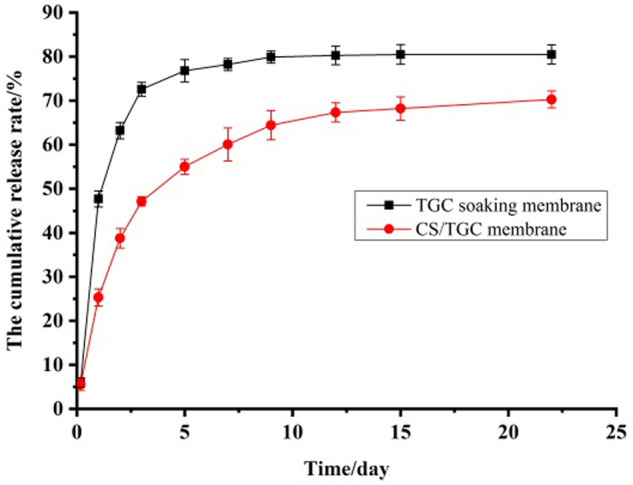
Drug release from biologic antimicrobial materials in static release mode. The experiments were performed in triplicate

The *in vitro* release behavior of nanodrug-loaded antibacterial biofilm was investigated by static dialysis as shown in Fig. 4. The drug release of TGC was time dependent. There was a certain sudden release of TCG in the early release, and the drug release rate reached 25% within 1 day, and more than 40% in 3 days. This provided a guarantee for antimicrobial membrane to rapidly achieve the effective germicidal concentration. However, in the later stage, the drug release rate was obviously slow release, and the cumulative release rate could reach more than 75% after 22 days.

### Cytotoxicity evaluation of drug-loaded antimicrobial membrane

**Figure 5.**
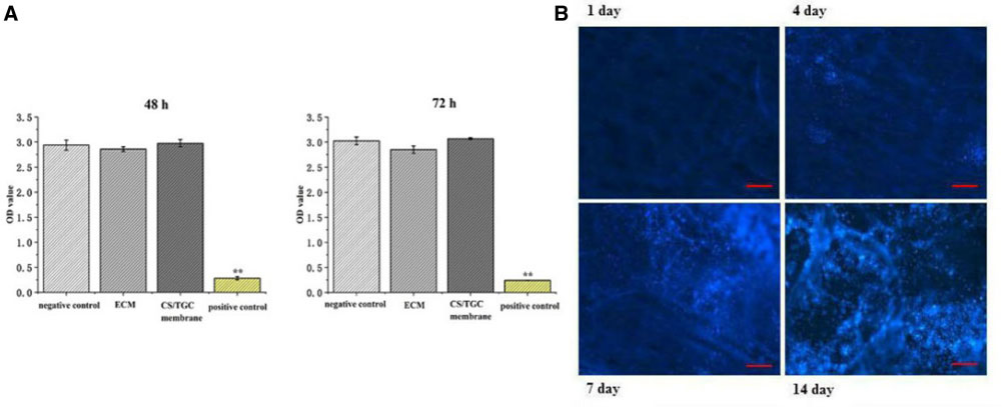
Cell toxicity evaluation of CS/TGC membrane. (A) The MTS assay of fibroblast L929 in different groups. (B) DAPI staining of fibroblast L929 in CS/TGC membrane in different times. Bar = 200 µm, **P <* *0.01

The supernatant of the immersed antimicrobial membrane was collected to determine the toxicity to L929 cells with MTS assay kit. Our results showed that there was no significant toxicity of biological membrane immersed medium in 48 and 72 h (Fig. 5A).

On the fourth and seventh day, there was significant cell proliferation on the membrane. DAPI staining also showed that the number of cells in the antimicrobial biofilm increased significantly on day 14 (Fig. 5B).

### *In vivo* compatibility of drug-loaded antimicrobial membrane

**Figure 6.**
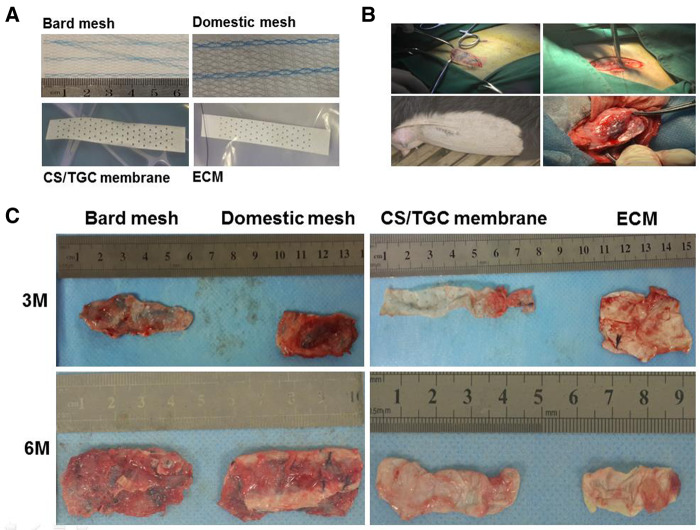
The evaluation of compatibility *in vivo*. (A) Appearance of the figures of different materials. (B) The process of implantation under the abdominal wall. (C) The gross figures of membrane in different times

Bard mesh, domestic mesh, drug-loaded antimicrobial membrane and pure ECM membrane were implanted under the abdominal wall of goats as shown in Fig. 6A. Bard is a porous mesh membrane woven from polypropylene, which is commonly used in pelvic floor repair material in clinic. In the study, we used it as a control. In order to investigate the biocompatibility *in vivo*, these four membranes were implanted under the peritoneum. The operation process is shown in Fig. 6B. The pictures of the specimens taken out at 3 and 6 months after implantation are shown in Fig. 6C and D. Bard mesh implantation triggered less tissue inflammation in the early stage and had more scar absorption at 6 months postoperatively, whereas the domestic mesh was more likely to cause scar thickening and excessive tissue growth.

In our drug-loaded antimicrobial membrane group, there was no serious tissue agglomeration and the membrane maintained a good spreading structure. The surrounding tissues began to grow through the pores and channels of the biological membrane. After operation of 6 months, 1-mm macropores of the biological membrane were occupied and replaced by new tissues.

**Figure 7.**
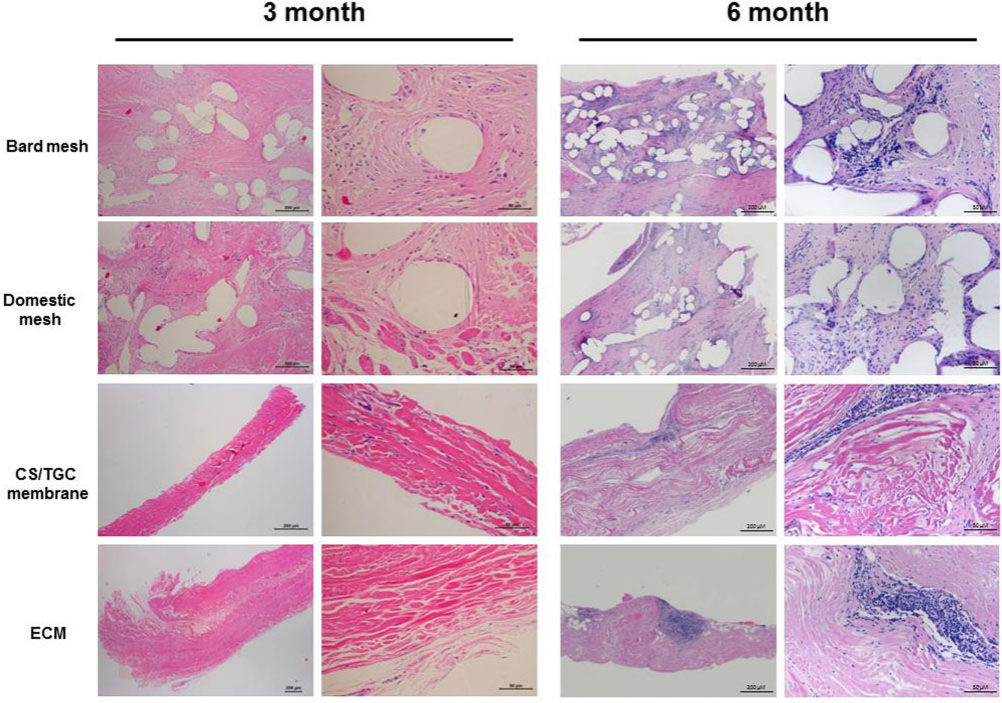
Histopathologic evaluation of CS/TGC membrane *in vivo.* HE staining of different groups after 3 and 6 months

HE staining of histopathological specimens 3 and 6 months after implantation is shown in Fig. 7. A small amount of infiltrated lymphocyte (<25/High power field (HPF)), infiltrated multinucleated giant cell (1–2/HPF) and some capillaries (4–7/HPF) were found in each group. The four membranes did not show significant histological differences 3 months after operation (Fig. 7). The results of HE staining at 6 months after operation showed that there were more lymphocyte infiltration (26–50/HPF) and more capillaries (1–3/HPF). The compatibility between the materials and the surrounding tissues was good. There was no obvious gap between the original membrane and the surrounding tissues. The structure of collagen fibers was intact, and the arrangement of fibers kept in a good order. A small amount of new fibrous tissue and capillaries grew into the implanted membrane. In summary, pathological histological analysis showed that the four materials all maintained good biocompatibility and safety *in vivo*.

**Figure 8.**
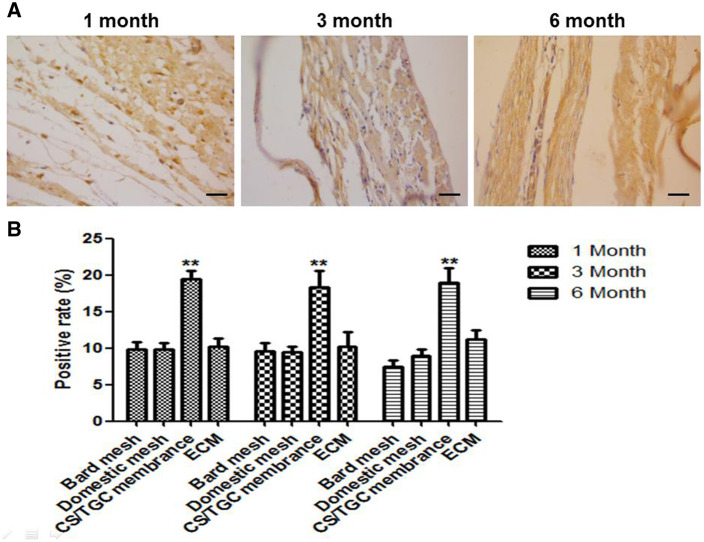
MMP9 expression in different groups. (A) The representative result of MMP9 expression in CS/TGC membrane in different times. (B) Quantitative analysis of MMP9 expression in different groups in different times. Bar = 200 µm, **P <* *0.01

The positive rate of MMP9 in the tissues around antimicrobial biological membrane was significantly higher than that of other three groups at 1, 3 and 6 months, indicating that the collagen fibers of the antimicrobial membrane began to rebuild and had a good biocompatibility (Fig. 8A and B).

### Antibacterial activity of drug-loaded antimicrobial membrane

**Figure 9.**
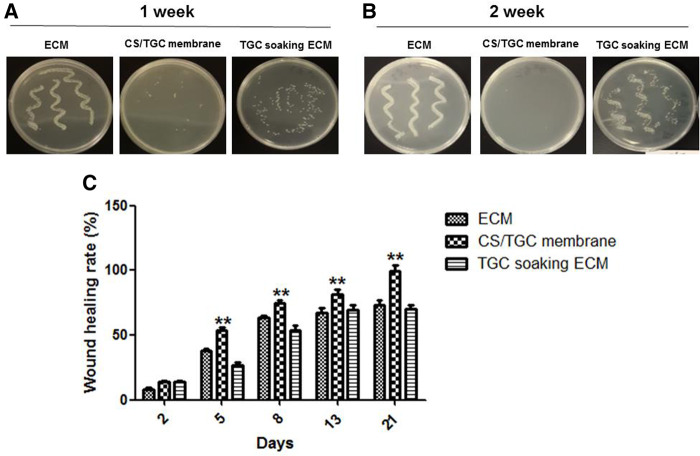
The evaluation of antibacterial ability in the CS/TGC membrane and the wound healing in CS/TGC membrane group. (A, B) The antibacterial test of membrane material after 1 and 2 weeks. (C) The wound healing in different groups. **P <* *0.01

The rabbits were sacrificed 1 week after implantation, and the membrane was taken out and inoculated on the agarose plate to observe the bacterial culture. Bacteria could grow from the antibiotic membrane, but the number of bacteria was significantly less than that of the blank membrane. The reason was that antibiotics could kill bacteria in a short time, but they could not be effective for a long time due to its half-life (Fig. 9A and B). When the concentration of antibiotics was lower than the sterilization concentration, the residual bacteria would grow again. The drug-loaded antimicrobial membrane could effectively maintain bactericidal for a long time, and the colony growth was significantly reduced. On the 21st day after operation, the wound healing rate of drug-loaded antimicrobial membrane group was close to 100%, whereas that of the other two groups was lower than 70%. The difference was significant (Fig. 9C).

## Discussion

The application of transvaginal mesh in pelvic floor repair was very common until FDA issued a warning [21]. In 2008, FDA issued the first public health notification on the complications of transvaginal mesh used in pop repair [22, 23]. In 2011, a safety communication was issued for the same reason of mesh complications [24, 25].

The pelvic meshes consisted of synthetic material mesh and biomaterial mesh [26]. In the past few decades, synthetic material meshes have been the main choice for pop surgery [27, 28], but they are associated with many complications, including erosion, infections, bleeding and chronic pain [29, 30]. Polypropylene (PPL) mesh is the most common synthetic material mesh because of its chemical stability and nonbiodegradable nature [31]. However, PPL mesh has the complications such as foreign body reaction, persistent inflammation and excessive fibrosis [32, 33].

Biomaterial meshes composed of mammalian ECM derived from porcine small intestinal mucosa, bovine pericardium have been used in POP surgery [31]. They often degrade rapidly and produce an intense inflammatory reaction leading to fibrosis [31, 34]. However, the mammalian ECM specially treated by the patent technology of chemically cross-linking (US 6106555) can effectively prolong the degradation cycle and promote tissue growth.

Bacterial infection is one of big challenges in the repair of pelvic floor, especially the use biomaterial meshes. In our study, TGC and CS were coated to the biomaterial mesh to improve its high efficiency and long-time bactericidal efficacy. Antimicrobial test and drug release test showed that drug-loaded antimicrobial membrane had strong antimicrobial activity against *E. coli* and *S. aureus*.

Compared with Bard mesh and domestic mesh membrane, CS/TGC antimicrobial biological membrane had good biocompatibility, cell affinity and less inflammation, which ensured the safety of implantation *in vivo*. In order to further study the possible process of collagen degradation in membranes, MMP9 was detected by immunohistochemistry. MMP9 belongs to the matrix metalloproteinases family and is a kind of gelatinase [35, 36]. Our study found that the expression of MMP9 in our antimicrobial membrane was high, which indicated that the collagen fiber was degraded and the biocompatibility was good.

In addition, the rabbit model of subcutaneous infection on the back showed that our biological membrane had a good long-term antibacterial effect and could enhance the ability of animal tissue repair.

## Conclusion

In this study, we successfully fabricated a novel CS/TGC antimicrobial biological membrane that exhibited good biocompatibility, low toxicity and strong antibacterial ability. The results of this study showed that this new antibacterial membrane is a promising membrane for pelvic floor tissue repair and functional reconstruction.

*Conflict of interest statement*. None declared.
